# Radioimmunotherapy of experimental head and neck squamous cell carcinoma (HNSCC) with E6-specific antibody using a novel HPV-16 positive HNSCC cell line

**DOI:** 10.1186/1758-3284-3-9

**Published:** 2011-02-12

**Authors:** Matthew Harris, Xing Guo Wang, Zewei Jiang, Gary L Goldberg, Arturo Casadevall, Ekaterina Dadachova

**Affiliations:** 1 Department of Nuclear Medicine, Albert Einstein College of Medicine, Albert Einstein Cancer Center, Bronx, New York, USA; 2 The Faculty of Life sciences, Hubei University, Wuhan, PR China; 3 Department of Obstetrics & Gynecology and Women's Health, Division of Gynecologic Oncology, Albert Einstein College of Medicine, Bronx, New York, USA; 4 Department of Microbiology and Immunology, Albert Einstein College of Medicine, Bronx, New York, USA; 5 Department of Medicine, Albert Einstein College of Medicine, Bronx, New York, USA

## Abstract

**Background:**

Head and neck squamous cell carcinoma (HNSCC) is the sixth most common malignancy worldwide with a poor prognosis. Human papilloma virus (HPV) infection is associated with 20% HNSCC, and 50% of oropharyngeal carcinoma. HPV16 type is detected in 90% of all HPV+ HNSCC. Recently we suggested a fundamentally different approach to treatment of cancers of viral origin by targeting viral antigens on cancer cells with radiolabeled antibodies (mAbs) which promises exquisite specificity of treatment. We aimed at extending this approach to HPV-related head and neck cancer by performing radioimmunotherapy (RIT) targeting E6 and E7 oncogenes with radiolabeled mAbs.

**Methods:**

We first aimed at developing HPV16+ cell line and animal model for RIT of HNSCC as at present there are no commercially available HPV16+ HNSCC cell lines and there is only one HPV+ cell line among the collection maintained by Dusseldorf, Michigan and Turku groups. Commercially available HNSCC cell line FaDu was transfected with pLXSN16E6E7 vector containing HPV16 E6 and E7 genes. Generated novel cell lines were evaluated by PCR and western blot and the tumorigenecity was assessed in nude mice. Proof of principle RIT targeting E6 oncoprotein in 2A3 tumor-bearing nude mice was conducted using unlabeled or 188-Rhenium (^188^Re)-labeled C1P5 mAb to E6.

**Results:**

Novel HPV16+ 2A3 cell line reliably expressed E6 oncoprotein. E6 expression was modifiable with proteasome inhibitor MG132 in a dose-dependent manner. The levels of E6 expression in 2A3 cell line were estimated to be around 200 HPV copies per cell. The HPV16+ 2A3 cell line preserved 100% tumorigenicity of parent FaDu cells in nude mice. During RIT of 2A3 tumors in nude mice the relatively low dose of 200 μCi ^188^Re-C1P5 mAb was effective in decreasing the tumor growth in comparison with untreated controls. Unlabeled C1P5 mAb also caused some decrease in tumor progression, however, much less pronounced than ^188^Re-C1P5 mAb.

**Conclusions:**

We describe a proof-of-principle RIT study targeting HPV16 E6 oncoprotein with radiolabeled mAb to E6 in a stably transformed HPV16+ HNSCC cell line and tumor model in nude mice, and demonstrate potential utility of RIT as a novel molecular targeted therapy for HNSCC.

## Background

Oncogenic HPV infection is a causative factor in more than 90% of cervical cancers in women. It is also a risk factor for the development of penile and vulvar cancers. HPV infection is associated with a subset of head and neck squamous cell carcinomas (HNSCC), which are the sixth most common malignancy worldwide. HPV DNA is found in ~20% HNSCC, and ~50% of oropharyngeal carcinomas. HPV type 16 is the most prevalent and is found in 90% of all HPV positive HNSCC [1]. Oncogenicity in tumors caused by HPV is due to the prevalence of early genes E6 and E7 which disrupt normal cell growth and inhibit tumor-suppressor proteins. The viral oncoproteins E6 and E7 immortalize epithelial cells in culture and increase cellular transformation in concert with other oncoproteins [2-4]. E6 oncoprotein are located intracellularly and bind to p53, promoting its rapid degradation via the ubiquitin-dependent pathway, while E7 oncoprotein bind to the retinoblastoma (Rb) gene, thus causing ineffective cell growth regulation. As a consequence, the clinical outcomes for HPV-positive (HPV+) and HPV-negative (HPV-) head and neck cancers are often different [5].

**Figure 1:**
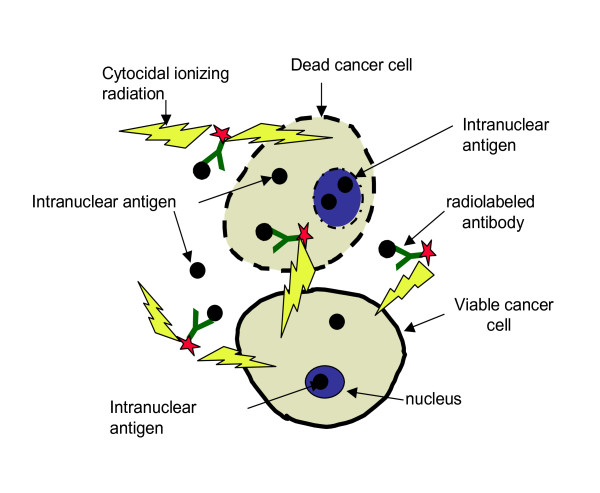
**Diagram detailing mechanism of HPV+ RIT with radiolabeled mAbs to intracellular antigens E6 and E7**. Intracellular antigens E6 and E7 are released from cancer cells as a consequence of a cellular turnover in a fast-growing tumor. E6 or E7-specific mAbs bind to extracellular E6 and E7 and deliver cytotoxic radiation to the area. The viable tumor cells including those with no or weak E6 or E7 expression are killed by radiation through "cross-fire" effect by radiation emitted in 360° sphere.

Radioimmunotherapy (RIT) uses tumor antigen-specific monoclonal antibodies (mAbs) for targeted delivery of cytocidal ionizing radiation to the tumor cells [6]. Radiolabeled mAbs have been approved for the treatment of primary, recurrent or refractory non-Hodgkin lymphoma (NHL). Our laboratory is interested in developing new therapies for HPV-related cancers based on RIT targeting E6 and E7 oncogenes with the radiolabeled mAbs [7]. Previously we showed the feasibility of targeting E6 oncoprotein in experimental cervical cancer by using radiolabeled mAb to E6 as a selective mediator of tumor destruction [8-10]. Though E6 and E7 oncoproteins are intranuclear and are not accessible to the mAbs in viable tumor cells - in fast growing tumors cellular turnover releases E6 and E7 oncoproteins into the extracellular space where they can be targeted for delivery of cytotoxic radiation by radiolabeled E6 or E7-binding mAbs via so called "cross-fire" effect (radiation emanating from one cell hitting the distant cell) (Figure 1). Naturally, for the development of RIT of HPV+ HNSCC it is important to have well characterized HPV+ HNSCC cell lines. However, at present there are no commercially available HPV+ HNSCC cell lines. Among the 29 HNSCC cells lines described in [11] such as cell lines maintained by the University of Michigan, University of Turku and University of Dusseldorf groups - only one cell line UD-SCC 2 is listed at HPV+ [11]. In this study we describe the generation of a stably transformed HPV16+ positive HNSCC cell line derived from the widely used and commercially available cell line, FaDu [12]. With this novel cell line we established a mouse model of HPV-associated head and neck cancer and conducted proof of principle RIT experiments with radiolabeled E6-specific mAb.

## Materials and methods

### Cell lines, Antibodies, and Reagents

FaDu and PA317 LXSN 16E6/E7 cell lines were procured from American Type Culture Collection (ATCC, Manassas, VA) and grown in DMEM media containing 10% Fetal Bovine Serum (Fisher) and 1% penicillin-streptomycin solution (penicillin, 10,000 U; streptomycin, 10 mg/mL; both from Fisher) at 37°C in a 5% CO_2 _incubator. The cell lines FaDu and PA317 were screened for mycoplasma by the manufacturer, ATCC, using both the Hoechst DNA stain and direct culture method. The antibiotics amount in the cell culture medium is recommended by ATCC and is considered inconsequential for cellular responses. The FaDu line was established in 1968 from a punch biopsy of a hypopharyngeal tumor. PA317 LXSN 16E6/E7 is a packaging cell line developed by transfection of the retrovirus vector pLXSN16E6/E7 into the Psi-2 ecotropic packaging cell line. The pLXSN16E6E7 vector contains the human papilloma virus (HPV) type 16 E6 and E7 genes under control of the Moloney murine leukemia virus (MoMuLV) promoter-enhancer sequences. The vector also contains a gene encoding resistance to neomycin transcribed from the SV40 promoter. This line produces the amphotropic retrovirus LXSN16E6/E7 encoding the HPV16 E6 and E7 open reading frames, which have been used to stably infect and immortalize many cell types. Cervical carcinoma HPV16-positive CasKi cell line (ATCC) was grown as in [6]. Murine anti-HPV16-E6, C1P5 antibody (IgG1 isotype) was obtained from Abcam (Boston, MA). Proteasome inhibitor MG-132 was purchased from Calbiochem (San Diego, CA) and BD Matrigel Basement Membrane Matrix - from BD Biosciences (Rockville, MD). The beta-emitter ^188^Re with a half life of 16.9 hours was produced from beta decay of 188-Tungsten parent (half life 69 days) using a ^188^W/^188^Re generator (Oak Ridge National Laboratory, Oak Ridge, TN). After ^188^Re was eluted in the form of sodium perrhenate, the C1P5 mAb was labeled "directly" with ^188^Re through binding of reduced ^188^Re to the generated -SH groups on the mAb as previously described [8-10].

### Transfection of FaDu cells with HPV16 Oncogenes E6 and E7

Transfection of the FaDu cell line with the HPV16 oncogenes E6 and E7 of PA317 LXSN16E6/E7 was performed by growing PA317 LXSN16E6/E7 cells in complete media until ~85% confluence. The media was changed and 16 hours after incubation with fresh media, the media containing the virus was removed and passed through a 0.4 μm low protein binding filter to remove cells and debris. The supernatant containing the virus was kept for further use. The FaDu cells were incubated under the same conditions until ~60% confluence. One mL of viral supernatant was combined with 3 mL of serum-free DMEM containing 4 μg/mL hexadimethrin bromide (polybrene) (Sigma) and overlaid onto the FaDu cells in 75 cm^2 ^flasks. After incubation for 3 hours, another 5 mL of serum-free DMEM containing 4 μg/mL polybrene was added to the flasks and incubated for another 4 hours. The media/viral supernatant mixture was then removed and 25 mL of complete DMEM was added to each flask, which were incubated for another 48 hours. Selection of transfected cells was done using the neomycin analogue, G418 (1 mg/mL) (Fisher), for 7 days. The surviving cells were then cultured with complete DMEM containing 200 μg/mL G418 for the duration of the project. Pure colonies were established by serially diluting the surviving cells in a 96-well plate. Wells containing only one cell were grown and eventually transferred to 75 cm^2 ^flasks for further propagation. The growth of the transfected cells was compared to that of FaDu cells for 6 days and doubling times for both cell lines were calculated using the following formula:

Doubling time=ln2/(ln(A/Ao)/t), where A−amount of cells at time t, Ao–amount of cells at time 0.

### DNA Extraction and PCR

Polymerase Chain Reaction (PCR) was used to determine the presence of oncogenes E6 and E7 in the transfected cell lines. DNA from the colony purified line was extracted and purified using the DNeasy Blood & Tissue Kit obtained from Invitrogen. The concentration of DNA was measured spectrophotometrically at 260 nm wavelength using a Spectra Max 250 96-well plate reader. The following oligonucleotides (Invitrogen) were used: E6F1 5'-ATGTTTCAGGACCCACAGGA-3'; E6R1 5'-GGTTTCTCTACGTGTTCTTGA-3'; E7R1 5'-TCCAGCTGGACCATCTATTTC-3'. The E6 and E7 oncogenes overlap on the vector thus allowing for two products - E6 alone and E6 + E7. Platinum PCR SuperMix High Fidelity from Invitrogen was added to each PCR reaction tube along with 150 ng of genomic DNA, and 3.0 μL of primer (300 nM). The tubes were incubated initially at 94°C for 1 min to denature the template and to activate the enzyme followed by 40 temperature cycles of: 95°C for 30 seconds, 50°C for 40 seconds, 68°C for 40 seconds. DNA from CasKi cells which have high number (~ 600) of HPV16 copies per cell was used as positive control.

### Western Blotting

Western blotting was performed to establish the presence of E6 oncoproteins in the transfected FaDu cells as well as to estimate the level of E6 expression in this cell line in comparison with cervical cancer cell line CasKi. Cell pellets were suspended in the lysis buffer (4% SDS, 20% glycerol, 0.5 M TrisHCl (pH 6.8), 0.002% bromophenol blue and 10% b-mercaptoethanol). Protein samples were boiled in water for 5 min before running SDS-PAGE. Twenty five to thirty μl of protein solution was loaded into each well of 12% pre-cast SDS-PAGE gel (Bio-Rad). SDS-PAGE was used to monitor the relative protein amounts in the samples. All samples were normalized with a tubulin control. Twelve percent SDS-PAGE gel was used to separate proteins, and electrophoresis was performed using Mini-Protean^® ^3 Cell system (Bio-Rad). After electrophoresis, the gel was transferred into the PVDF transfer buffer (25 mM Tris, 190 mM glycine and 2.5% (v/v) methanol) for 5 min. Then, proteins were transferred from the gel to the Immun-Blot™ PVDF membrane (Bio-Rad) on Semi-dry Electrophoretic Transfer Cell (Bio-Rad) at 15 V for 17 min. The membrane was soaked in the blocking solution (5% non-fat dry milk in the 0.1% TBST) with gentle shaking for one hour. The membrane was incubated in TBST blocking solution (0.1% Tween-20, 25 mM TrisHCl (pH 7.6), 500 mM NaCl, and 5% non-fat dry milk) containing 1:2000 diluted anti-HPV16 E6 C1P5 mAb at 4°C overnight. After several washes with TBST, the membrane was hybridized by the secondary antibody conjugated with horseradish peroxidase (Rabbit polyclonal to mouse IgG (HRP)) in TBST blocking solution with a 1:10,000 dilution. After three washes with TBST, the membrane was incubated with Rodeo ECL detection reagents 1 and 2 (USB) for 5 min, and then exposed to CL-XPosure™ film (Pierce). The film was developed as per manufacturer's instructions. For estimation of E6 level in HPV transfected FaDu cells - the western blotts of this and of CasKi cell lines were subjected to ImageJ intensity quantification.

### Tumor Model

All animal studies were carried out in accordance with the guidelines of the Institute for Animal Studies at the Albert Einstein College of Medicine. 6-8 weeks-old athymic Nu/Nu CD1 nude mice were purchased from Charles River Laboratories. Ten million HPV transfected FaDu cells (3-4 passaging of cells) were mixed with 80% Matrigel and injected subcutaneously into the right flank of each mouse. The size of the tumors was measured every 3 days with calipers (Pro-max Sylvac System IP67, Fowler Tools and Instruments, Boston, MA) in three dimensions and the tumor volume was calculated as a product of three dimensions divided by 2. Electronic calipers help to ensure the precision of measurement and allow the immediate download of the measurements into the Excel file for tumor volume calculations, groups randomization etc.

### MG132 Treatment of Transfected FaDu Cells in vitro and in vivo

To study the response of HPV-transfected FaDu cells to MG-132 (Z-LLL-CHO, MW = 457.6) which is a potent, reversible and cell-permeable proteasome inhibitor (Ki = 4 nM) - the original and transfected FaDu cells were harvested and transferred into 4 sterile test tubes with complete media. Each tube contained 0.5 - 1 ml cell culture (cell concentration was ~10^6 ^cells/ml), and cells were allowed to grow at 37°C in 5% CO_2 _incubator overnight. Then, MG-132 solution was added to the tubes for the final concentrations of 0, 5, 10 or 25 μg/ml. The cells were incubated under the same conditions for another 3 hours. Finally, the cells were harvested by centrifugation, clarified by washing with PBS and processed for western blot as described above. For in vivo investigation of the MG-132 influence on the level of E6 expression in HPV-transfected FaDu tumors, 3 mice carrying tumors were injected intraperitoneally (IP) with 20 μg MG-132 and another group of 3 transfected tumor-bearing mice was used as control. Three hours later, all mice in both groups were sacrificed, their tumors removed, homogenized on ice and processed for western blot as described above.

### RIT of Transfected FaDu Tumors

To investigate the utility of the transfected FaDu cell line as a basis for an animal model for drug development, we conducted a proof of principle RIT study targeting E6 oncoprotein with ^188^Re-labeled C1P5 mAb. The mice with transfected FaDu tumors measuring 0.5-0.7 cm in diameter were randomized into groups of five. The groups were treated IP with either: 200 μCi ^188^Re-C1P5 mAb, matching amount (6 μg) of unlabeled ("cold") C1P5 mAb; or left untreated. The mice were observed for tumor growth and survival for 15 days. The experiment was performed twice.

### Statistical Analysis

Student t-test was performed to compare the doubling times for FaDu and transfected cell lines. The sample sizes in the animal experiments were pre-planned. The differences between the tumor sizes for differently treated groups in the RIT studies were analyzed by non-parametric Mann-Whitney test using Prism software (GraphPad, San Diego, CA). The differences were considered statistically significant when p-values were < 0.05.

## Results

### Generation and characterization of HPV16+ cell line

**Figure 2:**
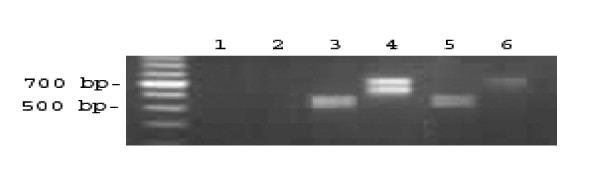
**PCR for HPV16 transfection**. Three cell lines were assessed at >P15 for both E6 and E7 oncogenes with total cellular DNA (lanes 1 and 2 - FaDu E6/E6E7, lanes 3 and 4 - CasKi E6/E6E7, lanes 5 and 6 - 2A3 E6/E7).

**Figure 3:**
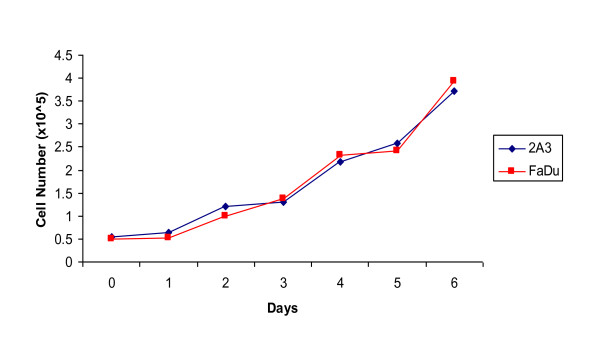
**In vitro growth analysis of both FaDu and the HPV transfected cell line, 2A3**. Approximately 5 × 10^4 ^cells were plated in a 6-well plate and grown at 37°C for 6 days.

**Figure 4:**
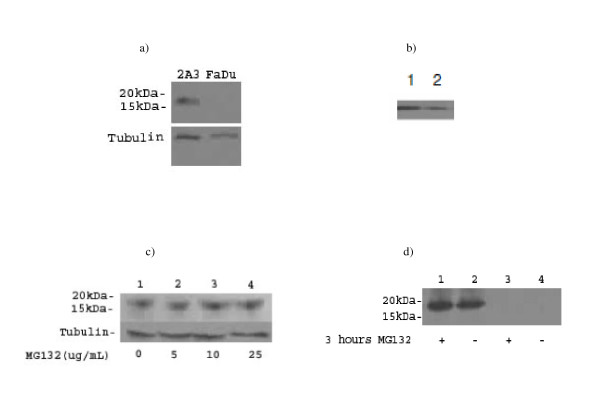
**Western Blot showing positive E6 expression from various protein extracts**. All samples were normalized with a tubulin control: a) 2A3 and FaDu cells grown in vitro; b) comparison of E6 levels in CasKi cervical cancer cells (lane1) and in 2A3 cells (lane 2). The intensity of the bands was compared using ImageJ software; c) 2A3 cells grown in vitro and treated with various concentrations of MG-132 for 3 hours. Lane 1-4 - 0, 5, 10 and 25 μg/ml MG-132, respectively; d) 2A3 and FaDu tumors in nude mice with and without treatment with 20 μg/ml of the proteasome inhibitor MG-132 3 hrs before extraction of the tumors (lanes 1 and 2 - 2A3, lanes 3 and 4 - FaDu).

After transfection a total of 10 new HPV16+ cell lines were produced. Cell line 2A3 was selected for further characterization as it most reliably expressed E6/E7 as demonstrated by PCR (Figure 2). Transfection was stable as the cells of >50 passages were still consistently expressing E6. Propagation the 2A3 cells in vitro demonstrated that they grew in culture in the same fashion as the parent cell line FaDu (Figure 3). Their doubling time of 51.8 hrs was no different from FaDu cells (48.1 hrs, P > 0.05). The 2A3 cells showed stable expression of E6 oncoprotein (Figure 4a). Comparison of E6 level in 2A3 using ImageJ quantification to that in cervical cancer CasKi cell line which has 600 HPV copies per cell revealed that amount of E6 in 2A3 cells should be equivalent to approximately 200 HPV copies per 2A3 cell (Figure 4b).

E6 levels in 2A3 cells were responsive to treatment with the MG-132 proteasome inhibitor, 10 μg/ml resulted in increased levels of E6 oncoprotein in the cells at 3 hrs post-treatment while the effect of the higher concentrations of MG-132 plateaued at 25 μg/ml (Figure 4c).

### In vivo characterization of HPV16+ 2A3 cell line

To test if the 2A3 cell line preserved the tumorigenecity of the parent FaDu cells in nude mice and continued to express E6 oncoprotein in vivo, we initiated xenografts in the flanks of female nude mice. The tumorigenecity rate of 2A3 cell line was 100% with 20 out of 20 mice developing tumors of 0.5-0.7 cm in diameter 10 days post inoculation with the cells. The tumors expressed high levels of E6 oncoproteins as demonstrated by western blot which were also increased up to 3 hrs post-treatment of tumor-bearing mice with MG-132 (Figure 4d).

**Figure 5:**
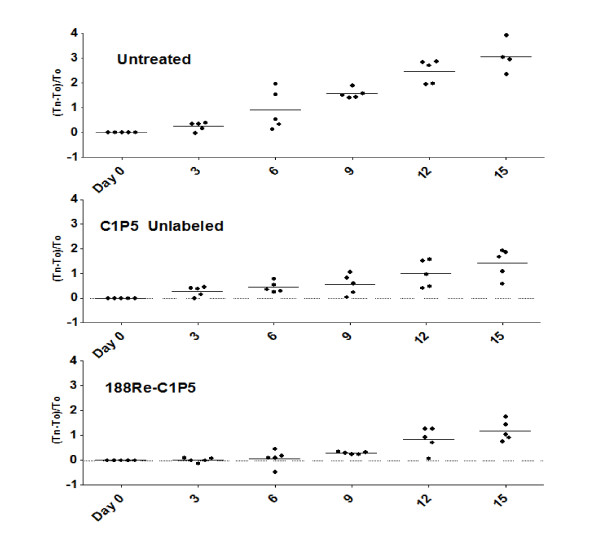
**Radioimmunotherapy of 2A3 tumors in mice**. Mice with tumors measuring 0.5-0.7 cm in diameter were randomized into groups of five. The groups were treated IP with either: 200 μCi ^188^Re-C1P5 mAb, matching amount (6 μg) of "cold" C1P5 mAb; or left untreated. Mice were observed for tumor growth and survival for 15 days. The experiment was performed twice. The differences in the tumor sizes between ^188^Re-C1P5 mAb and untreated group were significant (P < 0.05) for all time points; the differences in the tumor sizes between ^188^Re-C1P5 mAb and "cold" C1P5 mAb group were significant (P < 0.05) for Day 3-9 time points.

Since we are interested in developing mAbs-based therapies for head and neck cancers by targeting E6 or E7 oncoproteins with radiolabeled mAbs to these proteins, we conducted a proof of principle RIT study in 2A3 tumor-bearing mice with ^188^Re-C1P5 mAb, or matching amount of "cold" C1P5 mAb, and used untreated mice as controls (Figure 5). The relatively low dose of 200 μCi ^188^Re-C1P5 mAb was significantly effective in decreasing the tumor growth in comparison with untreated controls throughout the experiment (Table 1). Therapeutic effect of RIT was most apparent in the early time intervals studied. "Cold" C1P5 mAb also caused some decrease in tumor progression though not as dramatic as ^188^Re-C1P5 mAb - tumor regression was significantly more pronounced in RIT group than in "cold" mAb group on Days 3-9 post treatment (Table 1).

**Table 1:** P values for comparison of tumor sizes in ^188^Re-C1P5 mAb group to control groups in the RIT experiment.

Control group	Days post-treatment				
	
	3	6	9	12	15
**untreated**	**0.01**	**0.01**	**0.01**	**0.03**	**0.03**

**"Cold" C1P5**	**0.02**	**0.01**	**0.045**	0.08	0.10

The differences between the tumor sizes for differently treated groups were analyzed by non-parametric Mann-Whitney test. Statistically significant P values are in bold.

## Discussion

The HNSCC is characterized by a poor prognosis and novel, effective therapeutic options are urgently needed. A significant percentage of HNSCC's are HPV16+. The absence of a commercially available HPV16+ cell line hinders the research effort in developing new drugs for this disease. Though many laboratories cultivate their own HNSCC cell lines [11] - only one of them is reported to be HPV+. In addition, one fundamental and irrevocable requirement for the utilization of cell lines is their well-identified origin as well as the exclusion of cross-contamination by prokaryotic or eukaryotic cells. Thus, a straight-forward and reliable method of generating a HPV16+ HNSCC cell line using a commercial parent cell line and transfection reagents would be extremely useful in the field.

We generated a stably transformed HPV16+ cell line 2A3 derived from the widely used and commercially available cell line FaDu [12]. Expression of E6/E7 oncogenes in this novel cell line as determined by PCR was similar to that in patients when PCR was used [13]; from the comparison of western blot data with the well characterized cervical cancer cell line CasKi we estimated that 2A3 cell line has approximately 200 HPV copies per cell. Importantly for drug discovery research - this cell line was 100% tumorigenic in nude mice while continuing to express E6 oncoprotein in vivo.

Previous studies have shown that the proteasome inhibitor MG-132 reduces the degradation of ubiquitin-conjugated proteins in mammalian cells without affecting ATPase or isopeptidase activities. MG-132 has been reported to increase levels of E6 and E7 proteins in cervical cancer cells [14,15]. We also observed such increase in HPV16+ cervical cancer cells CasKi and SiHa in vitro and in vivo [8,9]. In this work we utilized the ability of E6 levels to respond to treatment with MG132 as a test to confirm the stable expression of E6 by the transfected cell line. Our 2A3 cell line was also responsive to treatment with MG132.

Our final aim was to perform the proof of principle RIT experiments targeting E6 in HNSCC with the radiolabeled mAb to this oncoprotein using novel HPV+ 2A3 cell line and animal model. Targeting of viral antigens within the tumors is fundamentally different from traditional RIT, which aims for cell surface associated tumor markers. The distinctive features of this approach are: 1) the targets are of viral origin as opposed to "self" human antigens, which minimizes cross-reactivity with host tissues, and 2) the viral proteins normally reside in intracellular compartments, such as the intranuclear location of the E6 and E7 oncoproteins. The significant decrease in tumor progression in mice with 2A3 tumors treated with low dose of ^188^Re-C1P5 mAb is encouraging as HNSCC is known to be resistant to external beam radiation therapy which in combination with chemotherapy is a standard treatment modality for this cancer [16]. Higher doses of radiolabeled mAbs to E6 or their repeated administration which we are planning to investigate in the future in the 2A3 animal model may have more long-lasting tumoricidal effects. In the control groups used in the proof of principle study "cold" mAb to E6 C1P5 also slowed down the tumor growth though not as pronounced as the radiolabeled mAb to E6. A similar effect on the tumor growth by the "cold" C1P5 was observed in experimental cervical cancer (8; Phaeton R. et al. unpublished observations) which was accompanied by complement deposition and invasion of inflammatory cells pointing at the involvement of complement dependent cytotoxicity (CDC) and antibody dependent cellular cytotoxicity (ADCC). The latter two have been recently observed in melanoma tumor bearing nude mice treated with "cold" antibody [17]. Thus, taken together these observations present attractive avenues for the development of molecular therapies for HNSCC such as RIT targeting viral antigens using the 2A3 cell line and animal model.

## Conclusions

We describe the proof of principle RIT experiments targeting E6 oncoprotein in HPV16+ experimental HNSCC which can provide a valuable new approach to treatment of HPV+ HNSCC. The novel stably transformed HPV16+ HNSCC cell line and animal model used in this study were generated using easy, reproducible method of transfection from the commercially available cell line FaDu.

## Competing interests

ED and AC are among co-inventors on the US patent application "Radioimmunotherapy and imaging of tumor cells that express viral antigens".

## Authors' contributions

All authors read and approved the final manuscript. ED designed the study; AC, XGW and GG contributed to the design of the study; MH, ZJ, XGW and ED performed the experiments and analyzed the results; ED, AC and GG wrote the paper.

## Authors' information

ED in collaboration with AC pioneered the use of radioimmunotherapy for treatment of infectious diseases as before it was exclusively a cancer treatment modality. They subsequently expanded this approach to treatment of cancers of viral origin. ED is an Associated Professor of Nuclear Medicine and Microbiology and Immunology at the Albert Einstein College of Medicine and is a Sylvia and Robert S. Olnick Faculty Scholar in Cancer Research.
